# Distribution of Cardiovascular Disease Risk Based on the Updated 2023 Guideline‐Recommended Australian Cardiovascular Disease Risk Algorithm and Comparison With the 2012 Algorithm: An Observational Study

**DOI:** 10.5694/mja2.70280

**Published:** 2026-09-02

**Authors:** Nina Lazarevic, Meghana Bhat, Grace Joshy, Danielle C. Butler, Mark Woodward, Anushka Patel, Rod T. Jackson, Garry Jennings, Rosemary Wyber, Ellie Paige, Emily Banks

**Affiliations:** ^1^ Australian National University Canberra Australian Capital Territory Australia; ^2^ The George Institute for Global Health, UNSW Sydney Sydney New South Wales Australia; ^3^ The George Institute for Global Health, Imperial College London London UK; ^4^ University of Auckland Auckland New Zealand; ^5^ Heart Foundation of Australia Melbourne Victoria Australia; ^6^ The Kids Research Institute Australia Perth Western Australia Australia; ^7^ QIMR Berghofer Medical Research Institute Brisbane Queensland Australia; ^8^ University of Queensland Brisbane Queensland Australia

**Keywords:** prevention and control, preventive medicine, primary care, risk assessment, risk factors

## Abstract

**Objectives:**

To quantify cardiovascular disease (CVD) risk, and implications for targeting preventive pharmacotherapy, using the 2023 guideline‐recommended Australian CVD risk algorithm, and compare this to the previous (2012) algorithm.

**Study Type:**

Application and comparison of two risk prediction algorithms.

**Setting and Participants:**

Data from 115,873 people aged 45–74 years without existing CVD, who had a clinical encounter between September 2020 and August 2022, recorded within MedicineInsight, a longitudinal primary care database covering 8% of Australian general practices.

**Main Outcome Measures:**

CVD risk distribution and risk categorisation into low‐, intermediate‐ and high‐risk groups under the 2023 and 2012 algorithms. Cohen's kappa and Bland–Altman plots were used to assess agreement and concordance.

**Results:**

Using the 2023 CVD risk algorithm and revised thresholds, 9.7% of participants were at high CVD risk (≥ 10% 5‐year risk or clinically determined high risk); 26.4% were at intermediate CVD risk (5% to < 10% 5‐year risk) and 63.9% were at low CVD risk (< 5% 5‐year risk). Corresponding 2012 figures for CVD risk were 17.6% high (> 15% 5‐year risk or clinically determined high risk), 11.6% intermediate (10%–15% 5‐year risk) and 70.8% low (< 10% 5‐year risk). Differences in proportions at high risk were largely driven by changes to clinically determined criteria for high risk. Overall, there was moderate‐to‐substantial agreement (linear‐weighted kappa = 0.62) and concordance (Kendall's tau‐*b* = 0.74) between algorithms.

**Conclusion:**

Proportions estimated at low risk and not routinely recommended pharmacotherapy align with international standards and were similar between guidelines. Although fewer people would be recommended pharmacotherapy due to being high risk under the updated versus previous guidelines, this likely reflects more accurate updated CVD risk estimation for the contemporary Australian population. The 2023 guidelines include a discretionary step (untested in our study) allowing adjustment based on additional factors. To ensure continued reduction of CVD burden across the population, we emphasise the use of this reclassification step by clinicians and consideration of the benefits of pharmacotherapy for those at intermediate risk.

## Plain Language Summary

**The Known:**

The 2023 Australian cardiovascular prevention guidelines replaced the 2012 guidelines. They recommend preventive medication for people at high (≥ 10%) 5‐year risk of a cardiovascular event and treatment consideration for those at intermediate (5% to < 10%) risk.

**The New:**

We found that 9.7% of people were at high cardiovascular risk, 26.4% were at intermediate cardiovascular risk, and 63.9% were at low cardiovascular risk under the 2023 guidelines, using primary care data for 115,873 people, compared with 17.6% at high risk, 11.6% at moderate risk and 70.8% at low risk under the 2012 guidelines.

**The Implications:**

Clinicians should discuss preventive medication use with people at intermediate cardiovascular risk, with treatment strongly considered, particularly for those with risk‐enhancing factors.

## 1 Introduction

Cardiovascular disease (CVD), predominantly coronary heart disease and stroke, is a leading and highly preventable cause of death worldwide and in Australia [1]. Interventions such as blood pressure‐lowering and lipid‐modifying therapies substantially reduce CVD risk and are World Health Organization ‘Best Buys’ [2, 3, 4]. In people without CVD, Australian and international primary prevention guidelines recommend preventive treatment according to CVD risk, calculated using algorithms accounting for multiple risk factors and often including consideration of high‐risk clinical conditions [5, 6].

**FIGURE 1:**
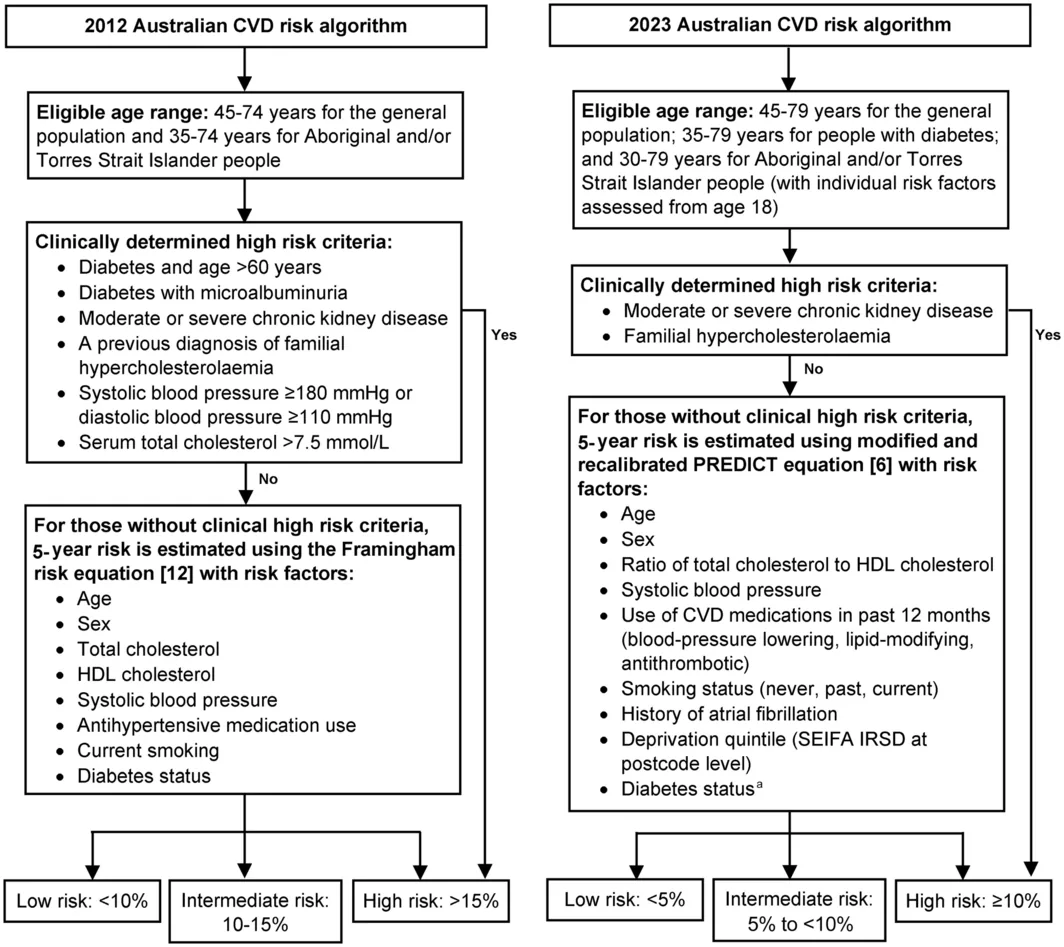
Eligibility, clinically determined high risk criteria and risk factors included in the 2023 and 2012 Australian cardiovascular disease risk algorithms. CVD, cardiovascular disease; HDL, high‐density lipoprotein; IRSD, Index of Relative Socio‐economic Disadvantage; SEIFA, Socio‐Economic Indexes for Areas. ^a^Diabetes‐specific equation with additional risk factors can be used for those with diabetes.

In 2023, new Australian primary CVD risk assessment and management guidelines were released, replacing the 2012 guidelines, including a three‐stage risk assessment algorithm, consisting of revised clinical high‐risk criteria, new CVD risk prediction equations with updated risk thresholds for initiating treatment and risk reclassification criteria enabling risk re‐categorisation based on additional risk‐modifying factors [5]. The new equations are based on contemporary New Zealand PREDICT equations [6, 7], re‐calibrated for the Australian population [8, 9, 10]. Previous 2012 guidelines incorporated more extensive clinical high‐risk criteria and used the United States Framingham risk equation [11, 12], known to overestimate risk in the overall Australian population while underestimating risk in First Nations people [8, 9, 13]. The 2023 equation (AUS‐PREDICT) includes additional risk predictors for area‐level socio‐economic deprivation, atrial fibrillation and CVD medication use [5]. There is also an optional Type 2 diabetes‐specific equation (AUS‐T2D‐PREDICT) with additional variables (Figure 1) [5].

Under the 2023 Australian guidelines, treatment with blood pressure‐ and lipid‐lowering medication is recommended for those at high 5‐year CVD risk (≥ 10%), with treatment considered for those at intermediate risk (5% to < 10%), depending on clinical context [14].

Given the differences between the two algorithms, use of the 2023 guidelines and treatment recommendations will likely result in a redistribution of individuals into low‐, intermediate‐ and high‐risk categories and changes in the allocation of preventive medications, compared with the 2012 algorithm. Quantification of the CVD risk distribution under the 2023 guidelines and comparison to previous guidelines is needed to understand the implications of implementing the 2023 guidelines in clinical practice and the potential impacts on population CVD burden.

The study aims were to quantify the CVD risk distribution under the 2023 guidelines in an Australian primary care cohort, estimate the proportions of individuals where treatment would be recommended (high risk), considered (intermediate risk) or not recommended (low risk) and compare these with classifications under the 2012 guidelines.

## 2 Methods

### 2.1 Data Sources

We used MedicineInsight, established by NPS MedicineWise (formerly the National Prescribing Service [NPS]) in 2011, containing longitudinal de‐identified individual‐level data, extracted from general practice clinical information systems [15]. MedicineInsight contains data from more than 3.2 million people, including sociodemographic data, prescribed medications, clinical measurements and pathology results from 662 participating general practices, accounting for 8.2% of Australian general practices [15, 16]. It is broadly representative of the Australian population based on age, sex, socioeconomic status and the proportion of people identifying as Aboriginal and/or Torres Strait Islander [16].

### 2.2 Study Population

We used a 25% random sample of people in MedicineInsight who had at least one non‐administrative clinical encounter between September 2020 and August 2022. We excluded people who had documented prior history of CVD (including carotid artery stenosis, coronary heart disease, heart failure, peripheral vascular disease, renal artery stenosis, stroke and transient ischaemic attack) or gestational diabetes during the study period [17].

### 2.3 Risk Algorithms and Outcomes

We conducted a head‐to‐head comparison of CVD risk estimated using the 2023 and 2012 risk algorithms, including the different thresholds for categorising risk (Figure 1) [5, 11].

Outcomes were estimated CVD risk and the proportion of people in each CVD risk category (low, intermediate and high). For risk assessment using the 2023 algorithm, we used AUS‐T2D‐PREDICT for people with Type 2 diabetes who had additional diabetes‐specific data and the general AUS‐PREDICT otherwise. To assess the impact of using the diabetes‐specific equation, we compared CVD risk using AUS‐PREDICT and AUS‐T2D‐PREDICT among those with complete diabetes‐specific data. In a supplementary analysis, we applied AUS‐PREDICT to the full study population.

### 2.4 Risk Factors

Risk factor definitions and missing data approaches are detailed in Table S1. We used risk factor measurements from the most recent clinical encounter (‘reference visit’), with retrospective time windows for capturing prescribed medications, clinical measures and pathology measures, varying 1–5 years, aligned with those used in the integrated 2023 CVD risk calculator (Table S1).

People with missing sex, age, smoking status, systolic blood pressure or cholesterol data were excluded. For the remaining risk factors, an absence of a specific diagnosis, medication prescription or pathology result was assumed to indicate that the patient did not have that disease or was not prescribed that medication. For deprivation quintiles (Socio‐Economic Indexes for Areas (SEIFA) Index of Relative Socio‐economic Disadvantage [IRSD] 2016) [18], we imputed missing values with mean levels.

### 2.5 Statistical Analysis

We first identified people automatically considered at high risk based on existing clinical conditions. We then estimated 5‐year primary CVD risk and categorised participants as low, intermediate or high risk using the risk equations and treatment thresholds recommended within each guideline [5, 11]. To align with the recommended age range in the 2012 guidelines, we included people aged 45–74 years in the main analysis and conducted a supplementary analysis applying the 2023 algorithm to all those eligible under the 2023 guidelines (ages 45–79 years, 30–79 years for Aboriginal and Torres Strait Islander people, 35–79 years for people with diabetes).

After combining high risk and clinically determined high‐risk categories, we used a linear‐weighted kappa statistic to assess agreement between risk classifications under the two algorithms. To assess agreement and quantify differences in risk estimates, we conducted a Bland–Altman analysis of the differences versus means of the risk estimates from the 2023 and 2012 equations [19]. We used Kendall's tau‐b statistic to quantify discordance between risk estimates. Analyses were performed in R v4.3.0.

Our study is reported according to the Strengthening the Reporting of Observational Studies in Epidemiology (STROBE) guidelines [20] (Table S2).

### 2.6 Ethics Statement

MedicineInsight approved the study (2021‐003), with ethics approval from the Australian National University Human Research Ethics Committee (2021/424) and the Human Research Ethics Committee of the Aboriginal Health and Medical Research Council of New South Wales (1730/20).

## 3 Results

### 3.1 Sample Characteristics

The study sample consisted of 115,873 people (Figure S1), with 63,182 female participants (54.5%), a median age of 59 years (interquartile interval [IQI], 52–66 years) and 101,227 participants (87.4%) who never or previously smoked (Table 1). Few people had moderate‐to‐severe chronic kidney disease (1228/115,873; 1.1%), familial hypercholesterolemia (361/115,873; 0.3%) or atrial fibrillation (3007/115,873; 2.6%). Within 12 months before the reference visit, 41,502/115,873 (35.8%) had been prescribed blood pressure‐lowering medications, 15,507/115,873 (13.4%) had been prescribed lipid‐modifying medications, and 5242/115,873 (4.5%) had been prescribed antithrombotic medications. Overall, 12,514/115,873 (10.8%) had Type 2 diabetes, with median 5 years since diagnosis (IQI, 2–11 years) and 1575/12,514 (12.6%) were prescribed insulin‐related medication (Table S3).

**TABLE 1:** Participant characteristics.

	Female	Male	Overall
Number of people	63,182	52,691	115,873
Age (years), median (IQI)	60 (52–67)	59 (52–66)	59 (52–66)
Age group (years)			
45–54	20,366 (32.2%)	17,566 (33.3%)	37,932 (32.7%)
55–64	22,688 (35.9%)	19,140 (36.3%)	41,828 (36.1%)
65–74	20,128 (31.9%)	15,985 (30.3%)	36,113 (31.2%)
Aboriginal and/or Torres Strait Islander person			
No or not recorded	61,972 (98.1%)	51,779 (98.3%)	113,751 (98.2%)
Yes	1210 (1.9%)	912 (1.7%)	2122 (1.8%)
Smoking status (2012 definition)			
Current smoking	8185 (13.0%)	8624 (16.4%)	16,809 (14.5%)
Non‐smoking	54,997 (87.0%)	44,067 (83.6%)	99,064 (85.5%)
Smoking status (2023 definition)			
Current smoking	7030 (11.1%)	7616 (14.5%)	14,646 (12.6%)
Past smoking	25,906 (41.0%)	23,645 (44.9%)	49,551 (42.8%)
Never smoked	30,246 (47.9%)	21,430 (40.7%)	51,676 (44.6%)
Chronic kidney disease, moderate to severe (2023 definition)			
No	62,561 (99.0%)	52,084 (98.8%)	114,645 (98.9%)
Yes	621 (1.0%)	607 (1.2%)	1228 (1.1%)
Chronic kidney disease, moderate to severe (2012 definition)			
No	62,459 (98.9%)	51,985 (98.7%)	114,444 (98.8%)
Yes	723 (1.1%)	706 (1.3%)	1429 (1.2%)
Familial hypercholesterolaemia			
No	62,950 (99.6%)	52,562 (99.8%)	115,512 (99.7%)
Yes	232 (0.4%)	129 (0.2%)	361 (0.3%)
Systolic blood pressure (mmHg), median (IQI)	130 (120–141)	135 (126–144)	133 (123–142)
Diastolic blood pressure (mmHg)			
Median (IQI)	80 (73–87)	81 (75–88)	80 (74–87)
Missing	37 (0.1%)	27 (0.1%)	64 (0.1%)
Total cholesterol (mmol/L), median (IQI)	5.4 (4.7–6.1)	5.1 (4.4–5.8)	5.2 (4.6–5.9)
Total to high density lipoprotein cholesterol ratio, median (IQI)	3.4 (2.9–4.2)	4.1 (3.3–4.9)	3.7 (3.0–4.5)
Use of blood‐pressure lowering medication			
No	42,151 (66.7%)	32,220 (61.1%)	74,371 (64.2%)
Yes	21,031 (33.3%)	20,471 (38.9%)	41,502 (35.8%)
Use of lipid‐modifying medication			
No	55,624 (88.0%)	44,742 (84.9%)	100,366 (86.6%)
Yes	7558 (12.0%)	7949 (15.1%)	15,507 (13.4%)
Use of antithrombotic medication			
No	60,837 (96.3%)	49,794 (94.5%)	110,631 (95.5%)
Yes	2345 (3.7%)	2897 (5.5%)	5242 (4.5%)
History of atrial fibrillation			
No	61,995 (98.1%)	50,871 (96.5%)	112,866 (97.4%)
Yes	1187 (1.9%)	1820 (3.5%)	3007 (2.6%)
Socioeconomic quintile (SEIFA IRSD, 2016) [18]			
1 (most disadvantaged)	10,970 (17.4%)	9320 (17.7%)	20,290 (17.5%)
2	13,012 (20.6%)	10,902 (20.7%)	23,914 (20.6%)
3	12,909 (20.4%)	10,872 (20.6%)	23,781 (20.5%)
4	11,784 (18.7%)	10,052 (19.1%)	21,836 (18.8%)
5 (least disadvantaged)	14,272 (22.6%)	11,307 (21.5%)	25,579 (22.1%)
Missing	235 (0.4%)	238 (0.5%)	473 (0.4%)
Diabetes mellitus Type 2			
No	57,368 (90.8%)	45,991 (87.3%)	103,359 (89.2%)
Yes	5814 (9.2%)	6700 (12.7%)	12,514 (10.8%)

Abbreviations: IQI, interquartile interval; IRSD, Index of Relative Socio‐economic Disadvantage; SEIFA, Socio‐Economic Indexes for Areas.

People with missing data had similar sex distribution, IRSD quintile distribution and proportion of Aboriginal and/or Torres Strait Islander people as complete cases (Tables S4 and S5). However, complete cases included more older people and more people with Type 2 diabetes (Table S4).

### 3.2 Comparison of CVD Risk Classification

#### 3.2.1 Overall Study Sample

**FIGURE 2:**
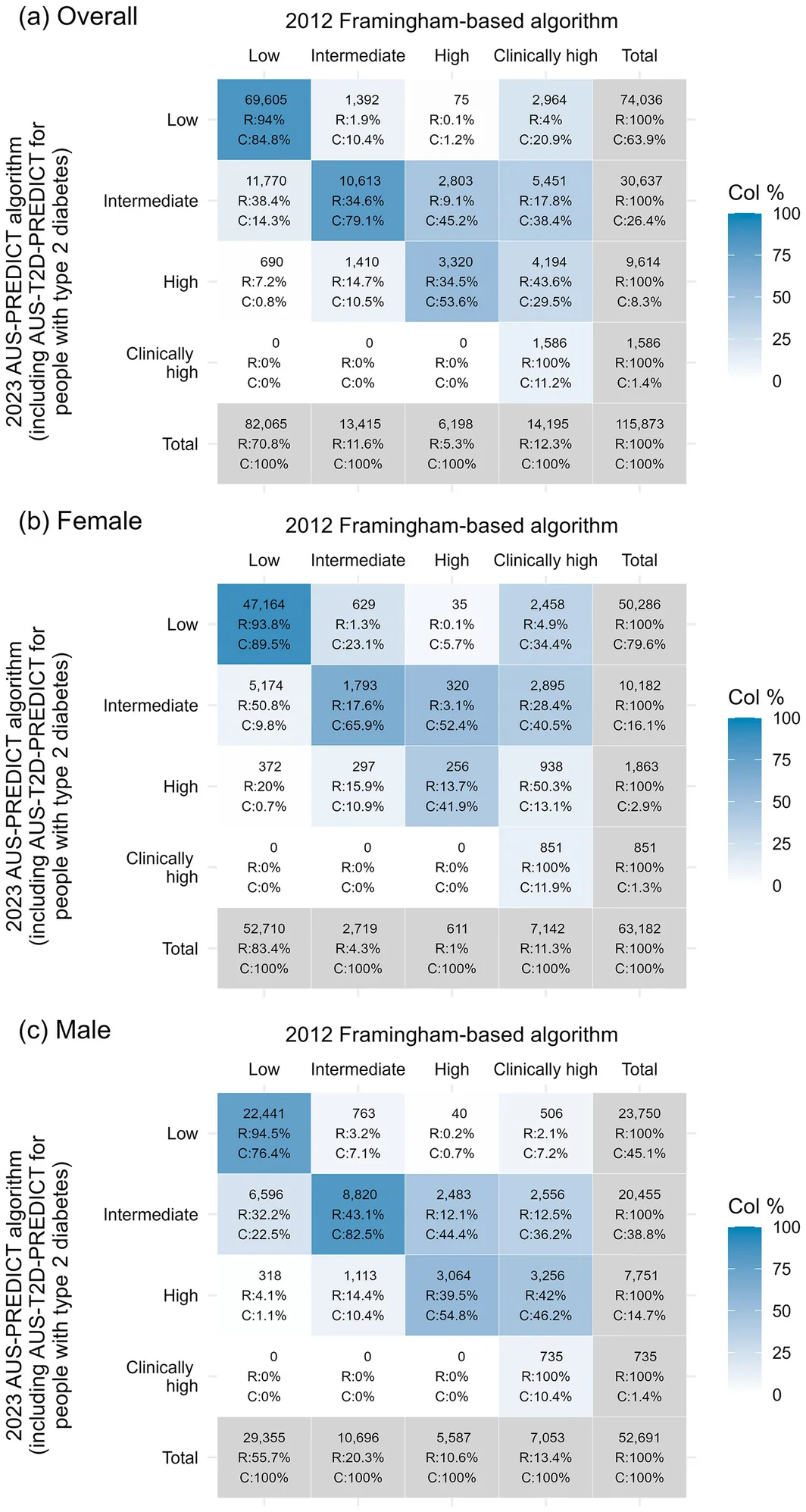
Risk classification under the 2023 and 2012 algorithms, overall and by sex, using AUS‐T2D‐PREDICT for people with type 2 diabetes and AUS‐PREDICT otherwise.^a^ AUS‐PREDICT, risk equation used in the 2023 Australian cardiovascular disease risk guidelines for the general population; AUS‐T2D‐PREDICT, risk equation used in the 2023 Australian cardiovascular disease risk guidelines for people with type 2 diabetes; C, column; R, row. ^a^Row and column percentages are shown beneath cell frequencies; blue cell shading reflects the column percentage.

**FIGURE 3:**
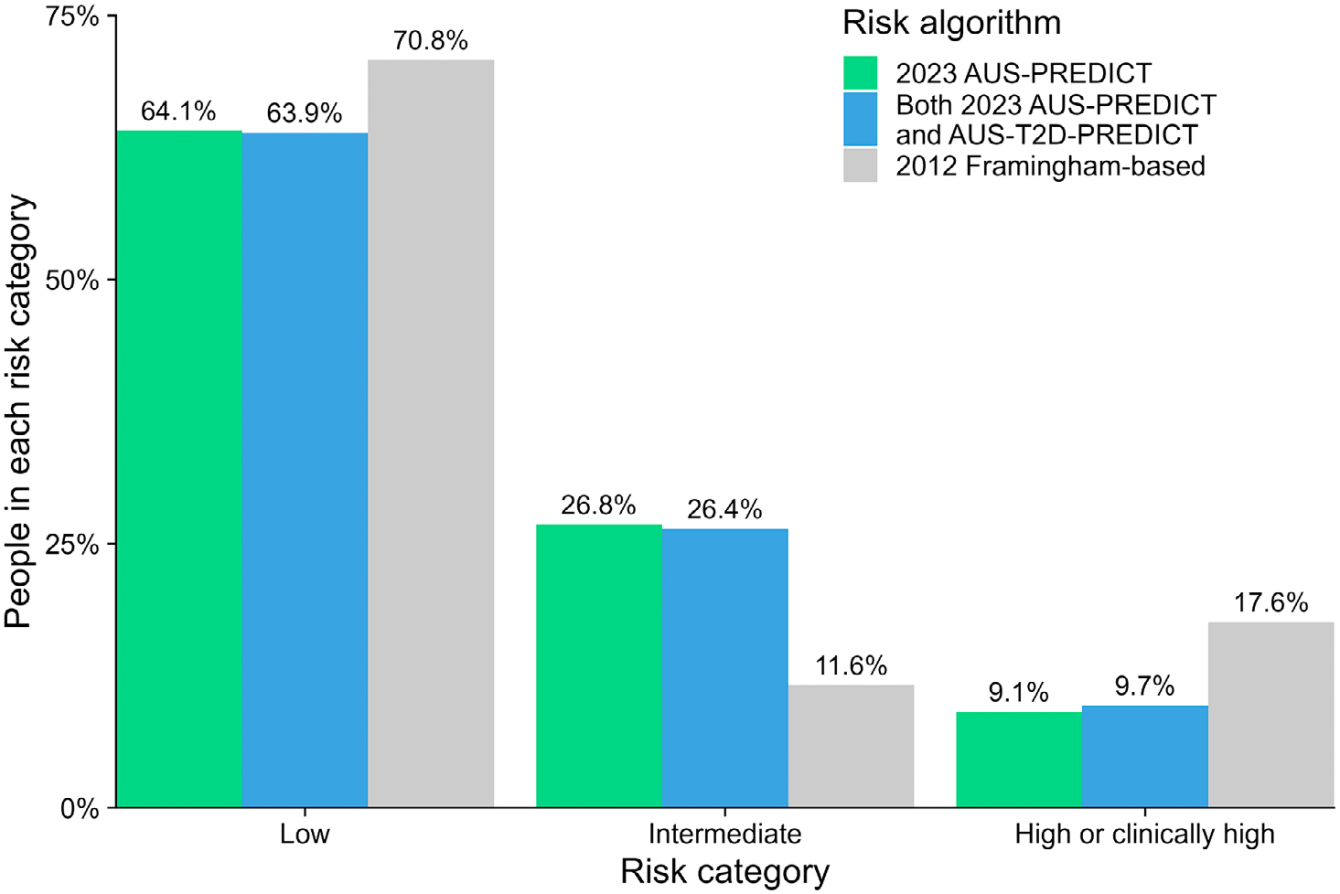
Risk classification under the 2023 and 2012 algorithms. AUS‐PREDICT, risk equation used in the 2023 Australian cardiovascular disease risk guidelines for the general population; AUS‐T2D‐PREDICT, risk equation used in the 2023 Australian cardiovascular disease risk guidelines for people with type 2 diabetes.

Under the 2023 algorithm, 11,200/115,873 (9.7%) of participants were classified as being at high CVD risk (comprised of 1586 [1.4%] at clinically determined high risk and 9614 [8.3%] at equation‐calculated high risk), 30,637/115,873 (26.4%) were classified as being at intermediate risk and 74,036/115,873 (63.9%) were classified as being at low risk. This compares with 20,393/115,873 (17.6%) at high risk (14,195 [12.3%] clinically determined high risk and 6198 [5.3%] at equation‐calculated high risk), 13,415/115,873 (11.6%) at intermediate risk and 82,065/115,873 (70.8%) at low risk under the 2012 algorithm (Figures 2a and 3). The pattern of greater proportion at intermediate risk with the updated algorithm was more pronounced at older ages (Figure S2). Similar proportions were at high, intermediate and low risk when the 2023 algorithm was applied to the broader age range recommended in the updated guidelines (Figure S3).

Of the people classified as being at low and high risk under the 2023 algorithm, 69,605/74,036 (94.0%) and 9100/11,200 (81.3%) were also classified as being at low and high risk, respectively, under the 2012 algorithm. For people classified as being at intermediate risk under the 2023 algorithm, 10,613/30,637 (34.6%) were classified as being at intermediate risk under the 2012 algorithm, whereas 11,770/30,637 (38.4%) and 8254/30,637 (26.9%) were classified as being at low and high risk, respectively, reflecting the pattern of movement to intermediate classification under the 2023 algorithm (Figure 2a).

Consistent with this, most people remained within the same risk category (89,318/115,873; 77.1%) or were classified in the adjacent lower (9646/115,873; 8.3%) or upper category (13,180/115,873; 11.4%) under the 2023 algorithm versus the 2012 algorithm (Figure 2a), largely driven by the broadly similar proportion at low risk. Overall, 3729/115,873 people (3.2%) moved up or down two categories, 2964/3729 (79.5%) of whom had clinically determined high risk under the 2012 algorithm but had their risk quantified under the 2023 algorithm. There was moderate‐to‐substantial risk classification agreement between the two algorithms (weighted kappa, 0.62 [95% confidence interval, 0.61–0.62]).

Differences in classifications were more pronounced for females; for example, of those at intermediate risk under the 2023 algorithm, 1793/10,182 (17.6%) were classified as being at intermediate risk and 5174/10,182 (50.8%) were classified as being at low risk under the 2012 algorithm (Figure 2b). Males were more likely to remain classified within the same risk category (Figure 2c). Results were unchanged when using the AUS‐PREDICT general equation for all people, irrespective of diabetes status (Table S6).

#### 3.2.2 Impact of the Type 2 Diabetes‐Specific Equation Under the 2023 Guideline

Of those with Type 2 diabetes, 7635/12,514 (61.0%) had additional diabetes‐specific data recorded. When using the diabetes‐specific equation compared with the general equation, most people remained within the same risk category (5502/7635; 72.1%) or were classified in the adjacent lower (638/7635; 8.4%) or adjacent upper (1459/7635; 19.1%) category, with only 36 participants (0.5%) moving across two categories (Table S7). Notably, for those classified as being at high risk using AUS‐T2D‐PREDICT, 977/2755 (35.5%) would have been classified as being at intermediate risk under AUS‐PREDICT.

#### 3.2.3 Comparison of CVD Risk Estimates Using the 2023 and 2012 Equations

Median CVD risk was 3.7% (IQI, 2.0%–6.2%) using the 2023 equations (AUS‐T2D‐PREDICT for people with Type 2 diabetes and AUS‐PREDICT otherwise) and 5.3% (IQI, 2.9%–8.8%) using the 2012 equation (Figure S4). Differences between risk estimate distributions were less pronounced for females than males (Figure S5).

The 2023 risk estimates were on average 2.3% lower in absolute terms (1.4% females, 3.3% males) and 34% lower in relative terms (geometric mean) (32% females, 36% males) than the 2012 risk estimates, with males more likely than females to have lower risk estimates under the 2023 guidelines versus the 2012 guidelines (Bland–Altman analysis, Figures S6–S8). With higher mean risk, absolute differences between the two risk estimates increased, but relative differences and variability decreased. Equations from the two guidelines were more likely to be concordant than discordant when ranking two people chosen at random in terms of their risk (Kendall's tau‐*b*, 0.74 [females, 0.69; males, 0.72]). For people with Type 2 diabetes, risk estimates from the 2023 diabetes‐specific equation were on average 1.3% higher in absolute terms and 12% higher in relative terms than from the general equation (Figure S9).

## 4 Discussion

In this large primary care population, around 10% of people were estimated as being at high CVD risk, for whom pharmacotherapy would be routinely recommended. A further quarter were at intermediate risk and would be considered for treatment, depending on clinical context, under the latest guidelines. Around two‐thirds would not be routinely recommended pharmacotherapy.

CVD assessment and management guidelines target preventive treatment to those at greater risk, while aiming to minimise overtreatment. There are currently large shortfalls in CVD preventive pharmacotherapy for people at high CVD risk [21]. Applying the updated algorithm, risks were, on average, 34% lower than under the 2012 algorithm, reflecting lower contemporary levels of CVD risk, with fewer people categorised as being at high risk and more categorised as being at intermediate risk. The 2023 guidelines recommend reclassification based on additional risk‐modifying factors as well as consideration of preventive medications for those at intermediate risk, depending on clinical context. Without reclassification, if only those at high risk were recommended treatment, 7.9% of the study population who would have been recommended treatment under the 2012 guidelines would not be universally recommended treatment under the 2023 guidelines, representing a 45% reduction in the number of people classified as high risk (11,200 vs. 20,393). This has implications for clinical management and impact on the population CVD burden. Given the larger proportion of people included in the intermediate risk category under the updated algorithm, our findings emphasise the need for treatment to be considered for those at intermediate risk, particularly people with risk‐enhancing factors and those with risk estimates close to the treatment threshold. Blood pressure‐ and lipid‐lowering medications lower the risk of future CVD events by 18%–25% each, are effective at all levels of risk and have favourable safety profiles [2, 3]. Clinician education about implementation of the 2023 guidelines should continue to emphasise consideration of reclassification and benefits of pharmacotherapy, in the context of individualised decision making, for people at intermediate risk.

Differences in CVD risk estimates and proportions of people categorised as being at low, intermediate and high risk in 2023 compared with 2012 were chiefly driven by changes in the risk algorithm and treatment recommendations. The 2023 equations were derived in a large, diverse, contemporary New Zealand cohort of more than 400,000 people [6, 7] and recalibrated for Australia [8, 9, 10]. The 2023 equation also includes additional risk factors, such as use of CVD medications, which influence risk. Newer medications (e.g., sodium–glucose cotransporter 2 [SGLT2] inhibitors and glucagon‐like peptide‐1 [GLP‐1] agonists) will likely be incorporated into future versions of the risk equations. In comparison, the 2012 equation was derived from a 1970s Framingham Heart Study cohort of about 5500 people [12] and was known to inaccurately predict risk for the Australian population.

The 2023 algorithm included fewer clinically determined high‐risk conditions than the 2012 algorithm, which rated individuals with common conditions such as diabetes with microalbuminuria as automatically at high risk. This change reflects variation in CVD risk among people with specific conditions, particularly diabetes. Recent data advances allow risk to be quantified within these broad categories. Overall, the proportion of people automatically assigned as being at high risk based on clinical criteria in the updated guidelines was around an eighth of that observed under the 2012 guidelines. This has important implications for risk communication and shared decision making. For example, it means that people aged over 60 years with well‐managed diabetes now have greater opportunity for individualised discussion of CVD risk and therapeutic options.

The reclassification stage of the 2023 risk algorithm gives clinicians the option to revise risk categories for people with additional risk‐modifying factors, where the risk equations may less accurately predict risk. It is recommended that clinicians consider upwards risk reclassification for: First Nations people, Māori people, Pacific Islander people, people of South Asian ethnicity, those with a family history of CVD, those without diabetes but with impaired renal function, those with an elevated coronary artery calcium score and people with severe mental illness. Although we were unable to assess the impacts of reclassification, if, for example, half those at intermediate risk under the 2023 algorithm were reclassified to high risk, then a larger proportion of people would be recommended preventive pharmacotherapy under the 2023 versus 2012 guidelines, resulting in greater reductions in CVD events across the population.

Finally, there was a lowering of the risk thresholds for categorising CVD risk in the 2023 Australian guidelines, aligning with international guidance [14] and counteracting, to an extent, the overall reductions in estimated risks under the 2023 algorithm.

This study is the first, to our knowledge, to quantify CVD risk and categorisation under the 2023 Australian CVD risk algorithm and to compare this to the 2012 algorithm. Strengths include: the use of a large‐scale primary care sample with general practice data from every state and territory in Australia; assessment and comparison of risk estimated using both clinically determined criteria for high risk and equations; and risk quantification using both the general and diabetes‐specific equations.

### 4.1 Limitations

Our study had some limitations. First, the performance of the 2023 risk equations has not been tested in the Australian population, and although the equations were recalibrated using Australian CVD event data, varying levels of miscalibration between the 2023 and 2012 risk equations will affect risk categorisation. The degree of miscalibration, overall and for specific groups (such as Aboriginal and Torres Strait Islander people), and impacts on risk categorisation should be assessed in future studies.

Second, we assumed that an absence of recorded disease diagnosis or medication prescription indicated a lack of that condition or medication. Although missingness is possible, lack of the condition or medication is more likely and is supported by findings of similar prevalence of health conditions and risk factors in our study sample compared with national estimates (data not shown).

Third, our study did not explicitly look at risk among First Nations people, who are at high risk of CVD earlier in life and have a greater CVD burden than non‐Indigenous Australians [22]. Comparison of the 2023 and 2012 CVD algorithms for First Nations people would be important to better understand changes in clinical practice and to address the inequitable burden of disease outcomes.

Finally, our findings are relevant to primary care populations—the target population for CVD risk assessment—and the absolute proportions reported here should not be generalised to the broader Australian population.

Although representing a substantial advance for Australia, the 2023 guideline‐recommended CVD risk equations are still based on equations derived from an overseas population. With an 11‐year gap between publication of the 2012 and 2023 CVD risk guidelines, consideration needs to be given to how guidelines can be more rapidly updated to reflect recent innovations in risk equation development, such as the recent publication of CVD risk equations developed using New South Wales primary care data [23].

## 5 Conclusions

The 2023 Australian CVD risk equations are likely to reflect contemporary risk more accurately than the previous equation, but their performance needs to be tested in the Australian population. Applying the updated risk thresholds, pharmacotherapy would be routinely recommended for around one‐tenth of this primary care population and considered for a further quarter, depending on clinical context. The major change is that more people will be classified as being at intermediate risk than at high risk. An unknown proportion of those people may be reassigned by clinicians to high risk based on reclassification factors. The implementation and public health implications of the reclassification factors, particularly for people classified as being at intermediate risk, require further consideration. Clinical decision making for people classified as being at intermediate CVD risk is emerging as a key determinant of the impact of the 2023 guidelines update on future CVD events at the population level.

## Author Contributions

**Nina Lazarevic:** writing (original draft), methodology, formal analysis, software, visualisation, writing (review and editing). **Meghana Bhat:** writing (original draft), software, visualisation. **Grace Joshy:** conceptualisation, methodology, writing (review and editing). **Danielle C. Butler:** writing (review and editing). **Mark Woodward:** methodology, writing (review and editing). **Anushka Patel:** writing (review and editing). **Rod T. Jackson:** writing (review and editing). **Garry Jennings:** writing (review and editing). **Rosemary Wyber:** writing (review and editing). **Ellie Paige:** conceptualisation, writing (original draft), visualisation, writing (review and editing). **Emily Banks:** conceptualisation, validation, writing (review and editing).

## Funding

Emily Banks, Anushka Patel and Rosemary Wyber are supported by the National Health and Medical Research Council (NHMRC) Investigator Grants (APP2017742, APP2016801 and 2025252, respectively). Ellie Paige is supported by a Future Leader Fellowship (107210) from the National Heart Foundation of Australia.

## Conflicts of Interest

Anushka Patel is a non‐remunerated Director and Chair of George Medicines, which has received investment and grants to develop fixed‐dose combination therapies for cardiovascular disease prevention. Emily Banks, Rod Jackson, Ellie Paige, Mark Woodward and Anushka Patel were members of the Algorithm Expert Subgroup that interpreted the evidence and made recommendations to the Guideline Expert Steering Group on aspects related to the risk equation and overall algorithm as part of the updating of the Australian guidelines on cardiovascular disease risk assessment and management. Emily Banks was the chair of the Algorithm Expert Subgroup and a member of the Guideline Expert Steering Group. Garry Jennings was co‐chair of the Guideline Expert Steering Group updating the Australian guidelines on CVD risk assessment and management. He is chief medical adviser to the National Heart Foundation of Australia.

## Supporting information


**Data S1:** Supplementary figures and tables.

## Data Availability

This study analysed the MedicineInsight dataset, which was originally established by NPS MedicineWise (formerly the National Prescribing Service) and is now managed by the Australian Commission on Safety and Quality in Health Care. The coefficients for the Framingham‐based risk equation used in the 2012 guidelines are available in Anderson et al. [12]. The coefficients for AUS‐PREDICT and AUS‐T2D‐PREDICT for estimating CVD risk using the 2023 guidelines are not publicly available but are available on application to the Heart Foundation of Australia (cvdriskteam@heartfoundation.org.au).
