# Supplementary material for: Distribution of Cardiovascular Disease Risk Based on the Updated 2023 Guideline‐Recommended Australian Cardiovascular Disease Risk Algorithm and Comparison With the 2012 Algorithm: An Observational Study
Source: Med J Aust. 2026 Sep 2;224(9):e70280. doi: 10.5694/mja2.70280 (PMC13537916; doi:10.5694/mja2.70280)
Supplement: Supplementary file 1 [file MJA2-224-0-s001.pdf]

Supporting Information

Supplementary material

This appendix was part of the submitted manuscript and has been peer reviewed. It is posted as supplied by the authors.

Appendix to: N. Lazarevic, M. Bhat, G. Joshy, et al. Distribution of Cardiovascular Disease Risk Based on the Updated 2023 Guideline-Recommended Australian Cardiovascular Disease Risk Algorithm and Comparison With the 2012 Algorithm: An Observational Study. *Medical Journal of Australia* 2026; doi: 10.5694/mja2.00000.

Table of Contents

Table S1: Risk factor definitions and treatment of missing values. 3

Table S2: STROBE checklist. 6

Figure S1: Flow chart outlining inclusions, exclusions, and final sample size. 9

Table S3: Summary statistics for diabetes-specific risk factors among people with type-2 diabetes. 9

Table S4: Distribution of selected risk factors among people who were not included in the analysis due to missing sex, smoking status, blood pressure, or cholesterol values. 10

Table S5: Distribution of selected risk factors among people who had missing and complete diabetes-specific risk factors among people with type-2 diabetes. 11

Figure S2: Risk classification under the 2023 and 2012 algorithms, by sex and age category. 12

Figure S3: Risk classification under the 2023 algorithms when including all people eligible (ages 45-79 years, 30-79 for Aboriginal and Torres Strait Islander people, and 35-79 years for people with diabetes). 13

Table S6: Risk classification under the 2023 and 2012 algorithms, overall and by sex, using the AUS-PREDICT general equation regardless of diabetes status. 14

Table S7: Risk classification under the 2023 algorithm for people with type-2 diabetes only, overall and by sex, comparing the AUS-PREDICT general equation and the AUS-T2D-PREDICT equation that includes additional (diabetes-specific) risk factors. 15

Figure S4: Distributions of cardiovascular disease risk estimates from the 2023 and 2012 risk equations. 16

Figure S5A and S5B: Distributions of cardiovascular disease risk estimates from the 2023 and 2012 risk equations, for females (A) and males (B). 17

Figure S6A and S6B: Bland-Altman plots of 2023 AUS-PREDICT (including AUS-T2D-PREDICT for patients with type-2 diabetes) versus 2012 Framingham-based cardiovascular disease risk estimates. 18

Figure S7A and S7B: Bland-Altman plots of 2023 AUS-PREDICT (including AUS-T2D-PREDICT for patients with type-2 diabetes) versus 2012 Framingham-based cardiovascular disease risk estimates for females. 19

Figure S8A and S8B: Bland-Altman plots of 2023 AUS-PREDICT (including AUS-T2D-PREDICT for patients with type-2 diabetes) versus 2012 Framingham-based cardiovascular disease risk estimates for males. 20

Figure S9A and S9B: Bland-Altman plots of cardiovascular disease risk estimates from AUS-T2D-PREDICT versus AUS-PREDICT for people with type-2 diabetes. 21

References 22

Table S1: Risk factor definitions and treatment of missing values.

<table>
<tr><th>Risk factor</th><th>2012 guideline</th><th>2023 guideline</th></tr>
<tr><td>Sex</td><td colspan="2">Within MedicineInsight, sex is defined as either biological sex or the category to which a person is assigned by themselves or others. People with missing sex were excluded from the analysis.</td></tr>
<tr><td>Deprivation quintile</td><td>N/A</td><td>We used the Australian Bureau of Statistics Index of Relative Socioeconomic Disadvantage (IRSD) 2016.[1] For two people who had a missing IRSD 2016 value but who had an IRSD 2011 value, we used the IRSD 2011 value. People with missing IRSD were set to the 2023 AUS-PREDICT equation mean deprivation levels, as per the AUS-PREDICT online calculator.</td></tr>
<tr><td>Diabetes status</td><td colspan="2">We used an existing conditions field for diabetes status in the MedicineInsight database. The diagnostic algorithm was developed by NPS MedicineWise using diagnosis data and reasons recorded for the clinical encounter and prescriptions. The algorithm has been validated for type 2 diabetes,[2] with sensitivity of 0.89. We assumed that missing health condition data indicated absence or no history of the condition.</td></tr>
<tr><td>Smoking status</td><td>Smoking status was defined using the latest clinician-recorded smoking status (current, ex-smoking, or non-smoking) and quit date where entered into the health record. For the 2012 risk equation, smoking status was defined as current smoking (including people who stopped smoking within 12 months of the reference visit) or non-smoking (including people who stopped smoking >12 months prior to the reference visit).</td><td>Smoking status was defined using the latest clinician-recorded smoking status (current, ex-smoking, or non-smoking) and quit date where entered into the health record. For the 2023 risk equation, smoking status was defined as current smoking, past-smoking (including people who had a quit date before the reference visit, unless recorded as a current smoker at the reference visit), or never smoking (non-smoking people with no recorded quit date).</td></tr>
<tr><td>Body mass index (BMI)</td><td>N/A</td><td>Weight (kg) divided by squared height (m), with weight limited to 35-250kg and height limited to 1-2.5m, and limited BMI to 15-60 kg/m^2. We used the most recently recorded BMI on or prior to the reference visit, with a maximum lookback of 2 years†. BMI was used only in the AUS-T2D-PREDICT equation, which defaulted to AUS-PREDICT if any risk factors were missing.</td></tr>
<tr><td>Systolic and diastolic blood pressure</td><td colspan="2">We used the average of the last two seated measurements on two separate occasions, on and prior to the reference visit, with a maximum lookback period of 2 years†. Observations of systolic blood pressure outside the range 30-300 mmHg, were set to missing. Individuals with missing systolic blood pressure data were not included in the analysis. For diastolic blood pressure, which is used only for determining 2012 clinical high risk, we assumed missing values meant lack of high diastolic blood pressure.</td></tr>
<tr><td>Total cholesterol, high-density lipoprotein (HDL) cholesterol, and the total:HDL cholesterol ratio</td><td colspan="2">We used the latest pathology test results within 1 month of the reference visit or up to 5 years† prior, ignoring fasting status. HDL cholesterol values <0.25mmol/L and total cholesterol <0.5mmol/L were set to missing.[3,4] Individuals with missing lipids data were not included in the analysis.</td></tr>
<tr><td>Urine albumin-creatinine ratio (uACR), microalbuminuria, persistent proteinuria/macroalbuminuria</td><td colspan="2">For <u>uACR</u>, we used the latest pathology test results within 1 month of the reference visit or up to 3 years† prior.
We defined <u>microalbuminuria</u> as uACR >2.5 mg/mmol for males and uACR >3.5 mg/mmol for females. We did not use the urinary albumin concentration to define microalbuminaria as it was not clear whether observations were spot samples or timed (24h) samples.
We defined <u>persistent proteinuria/macroalbuminuria</u>, as at least two positive test results within 3 months of the latest pathology test result associated with the reference visit. Positive results were defined as uACR >25 mg/mmol for males and uACR >35 mg/mmol for females.
Where uACR was missing, we assumed an absence of microalbuminuria. Where uACR was missing or was measured only once, we assumed an absence of persistent proteinuria/macroalbuminuria. For individuals with type 2 diabetes and</td></tr>
</table>

	missing uACR for use in the AUS-T2D-PREDICT equation, we used the AUS-PREDICT equation.	
Estimated glomerular filtration rate (eGFR), low eGFR, sustained low eGFR	We used the latest pathology test results within 1 month of the reference visit or up to 3 years[†] prior. We defined low eGFR as eGFR <45 mL/min/1.73m^2, and sustained low eGFR as at least two observations with eGFR <45 mL/min/1.73m^2, using the latest two tests.<br>Although the AUS-PREDICT guidelines specify the use of the CKD-EPI formula, the MDRD formula was also allowed as it was used in a substantial number of cases. Where eGFR was recorded as a categorical rather than continuous value, we used the limit as the observed value (e.g., we used eGFR of 90 where ≥90 was recorded).<br>Where eGFR was missing, we assumed an absence of low eGFR. Where eGFR was missing or was only measured once, we assumed an absence of sustained low eGFR. For individuals with type 2 diabetes and missing eGFR for use in the AUS-T2D-PREDICT equation, we used the AUS-PREDICT equation.	
Moderate-to-severe chronic kidney disease	Moderate to severe chronic kidney disease was defined as either persistent proteinuria/macroalbuminuria (as defined above), or low eGFR (for the 2012 guidelines) or sustained low eGFR (for the 2023 guidelines, as defined above), or recorded stage 3-5 chronic kidney disease. Where none of these health states were recorded in the data, we assumed that this indicated an absence or no history of moderate to severe chronic kidney disease.	
Glycated haemoglobin (HbA1c)	N/A	We used the latest pathology test results within 1 month of the reference visit or up to 3 years[†] prior.<br>We set to missing HbA1c values outside the ranges 9-195 mmol/mol and 3-20% as per the online calculator at the National Glycohemoglobin Standardization Program website (https://ngsp.org/ifcc.asp).<br>HbA1c was used only in the AUS-T2D-PREDICT equation, which defaulted to AUS-PREDICT if any risk factors were missing.
Familial hypercholesterolemia	We identified cases of familial hypercholesterolemia by searching free text fields on diagnosis, clinical encounter, and prescription reasons for versions of familial hypercholesterolemia, familial dyslipidaemia, or familial hyperlipidaemia. Where we were not able to identify cases based on the recorded data, we assumed that this indicated absence or no history of the condition.	
Atrial fibrillation	N/A	We used an existing conditions field for atrial fibrillation in the MedicineInsight database. The diagnostic algorithm was developed by NPS MedicineWise using diagnosis data and reasons recorded for the clinical encounter and prescriptions.[2]<br>We assumed that missing health condition data indicated absence or no history of the condition.
Lipid-modifying medications	Prescribed medications within one year[†] of the reference visit, including those specified in the 2023 guidelines:<br>pravastatin, simvastatin, atorvastatin, fluvastatin, ezetimibe, acipimox, bezafibrate, cholestyramine, clofibrate, colestipol hydrochloride, gemfibrozil, and nicotinic acid. Absence of these prescriptions was considered to indicate absence of lipid-modifying medication use.	
Blood-pressure lowering medications	Prescribed medications within one year[†] of the reference visit, including those specified in the 2023 guidelines:<br>angiotensin converting enzyme inhibitors (ATC codes C09A and C09B), betablockers (C07), thiazide (C03A), angiotensin II receptor blockers (C09C and C09D), and calcium channel blockers (C08). Absence of these prescriptions was considered to indicate absence of blood-pressure lowering medication use.	
Antithrombotic medications	Prescribed medications within one year[†] of the reference visit, including those specified in the 2023 guidelines: aspirin (acetylsalicylic acid), clopidogrel, ticagrelor, dipyridamole, prasugrel, ticlopidine hydrochloride, warfarin, warfarin sodium, phenindione, dabigatran, and rivaroxaban. Absence of these prescriptions was considered to indicate absence of antithrombotic medication use.	
Insulin	N/A	Prescribed medications within one year[†] of the reference visit, including insulins and analogues (ATC code A10A).<br>Absence of these prescriptions was

		considered to indicate absence of insulin use.
Prior history of documented CVD (including carotid artery stenosis, coronary heart disease, heart failure, peripheral vascular disease, renal artery stenosis, stroke, and transient ischaemic attack) (exclusion criteria)	We used an existing conditions field in the MedicineInsight database. The diagnostic algorithm was developed by NPS MedicineWise using diagnosis data and reasons recorded for the clinical encounter and prescriptions.[2] We assumed that missing health condition data indicated absence or no history of the condition.	
Gestational diabetes (exclusion criteria)	We used an existing conditions field in the MedicineInsight database to identify gestational diabetes within two years of the reference visit. The diagnostic algorithm was developed by NPS MedicineWise using diagnosis data and reasons recorded for the clinical encounter and prescriptions.[2] We assumed that missing health condition data indicated absence of the condition.	

† Retrospective time windows for capturing medications, clinical, and pathology measures were aligned with those used in the integrated 2023 CVD risk calculator (https://master.dr3cx4g8mtct5.amplifyapp.com/smart-app-launch.html).

Note: AUS-PREDICT and AUS-T2D-PREDICT refer to the risk equations used in the 2023 Australian cardiovascular disease risk guidelines for the general population and people with type-2 diabetes, respectively.

Table S2: STROBE checklist.

	Item No.	Recommendation	Page No. (submitted manuscript)	Relevant text from manuscript
**Title and abstract**	1	(*a*) Indicate the study's design with a commonly used term in the title or the abstract	**1**	**Observational study: application and head-to-head comparison of risk algorithms.**
		(*b*) Provide in the abstract an informative and balanced summary of what was done and what was found	**1**	**See Abstract.**
**Introduction**				
Background/rationale	2	Explain the scientific background and rationale for the investigation being reported	**2-3**	**See Introduction.**
Objectives	3	State specific objectives, including any prespecified hypotheses	**3**	**See last paragraph of Introduction.**
**Methods**				
Study design	4	Present key elements of study design early in the paper	**4**	**See Methods.**
Setting	5	Describe the setting, locations, and relevant dates, including periods of recruitment, exposure, follow-up, and data collection	**3-4**	**See Methods: Data sources, Study population, and Risk factors.**
Participants	6	(*a*) *Cohort study*—Give the eligibility criteria, and the sources and methods of selection of participants. Describe methods of follow-up<br>*Case-control study*—Give the eligibility criteria, and the sources and methods of case ascertainment and control selection. Give the rationale for the choice of cases and controls<br>*Cross-sectional study*—Give the eligibility criteria, and the sources and methods of selection of participants	**3-4**	**See Methods: Data sources, Study population.**
		(*b*) *Cohort study*—For matched studies, give matching criteria and number of exposed and unexposed<br>*Case-control study*—For matched studies, give matching criteria and the number of controls per case		
Variables	7	Clearly define all outcomes, exposures, predictors, potential confounders, and effect modifiers. Give diagnostic criteria, if applicable	**4, Supp. Table S1**	**See Methods: Risk algorithms and outcomes, Risk factors, with further details in Supp. Table S1.**

Data sources/ measurement	8*	For each variable of interest, give sources of data and details of methods of assessment (measurement). Describe comparability of assessment methods if there is more than one group	**4, Supp. Table S1**	**See Methods: Risk algorithms and outcomes, Risk factors, with further details in Supp. Table S1.**
Bias	9	Describe any efforts to address potential sources of bias	**N/A**	
Study size	10	Explain how the study size was arrived at	**Supp. Figure S1**	
Quantitative variables	11	Explain how quantitative variables were handled in the analyses. If applicable, describe which groupings were chosen and why	**4-5, Supp. Table S1**	**See Methods: Risk algorithms and outcomes, Risk factors, Statistical analysis, with further details in Supp. Table S1.**
Statistical methods	12	(*a*) Describe all statistical methods, including those used to control for confounding	**5**	**See Methods: Statistical analysis.**
		(*b*) Describe any methods used to examine subgroups and interactions	**5**	**See Methods: Statistical analysis.**
		(*c*) Explain how missing data were addressed	**4, Supp. Tables S1**	**See Methods: Risk factors, , with further details in Supp. Table S1.**
		(*d*) *Cohort study*—If applicable, explain how loss to follow-up was addressed<br>*Case-control study*—If applicable, explain how matching of cases and controls was addressed<br>*Cross-sectional study*—If applicable, describe analytical methods taking account of sampling strategy	**N/A**	
		(*e*) Describe any sensitivity analyses	**4**	**See Methods: Risk algorithms and outcomes, Statistical analysis.**
**Results**				
Participants	13*	(a) Report numbers of individuals at each stage of study—eg numbers potentially eligible, examined for eligibility, confirmed eligible, included in the study, completing follow-up, and analysed	**Supp. Figure S1**	
		(b) Give reasons for non-participation at each stage	**Supp. Figure S1**	
		(c) Consider use of a flow diagram	**Supp. Figure S1**	
Descriptive data	14*	(a) Give characteristics of study participants (eg demographic, clinical, social) and information on exposures and potential confounders	**5, Table 1, Supp. Table S2**	**See Results: Sample characteristics.**

		(b) Indicate number of participants with missing data for each variable of interest	**Table 1, Supp. Tables S2 & S4**	
		(c) *Cohort study*—Summarise follow-up time (eg, average and total amount)	**N/A**	
Outcome data	15*	*Cohort study*—Report numbers of outcome events or summary measures over time		
		Case-control study—Report numbers in each exposure category, or summary measures of exposure		
		Cross-sectional study—Report numbers of outcome events or summary measures	**6-7, Figure 2, Supp. Tables S5 & S6**	**See Results: Comparison of CVD risk classification.**
Main results	16	(*a*) Give unadjusted estimates and, if applicable, confounder-adjusted estimates and their precision (eg, 95% confidence interval). Make clear which confounders were adjusted for and why they were included	**5-7**	**See Results.**
		(*b*) Report category boundaries when continuous variables were categorized	**3, Figure 1 for CVD risk**	**See Methods: Risk algorithms and outcomes, with further detail in Figure 1.**
		(*c*) If relevant, consider translating estimates of relative risk into absolute risk for a meaningful time period	**7, Supp. Figures S6-S9**	**See Results: Comparison of CVD risk estimates using the 2023 and 2012 equations, and Supp. Figures S6-S9.**
Other analyses	17	Report other analyses done—eg analyses of subgroups and interactions, and sensitivity analyses	**6-7, Figure 2, Supp. Tables S5 & S6, Supp Figures S2, S5, S7-S9**	**See Results for analyses by sex, age group, and type 2 diabetes status.**
**Discussion**				
Key results	18	Summarise key results with reference to study objectives	**7-9**	**See Discussion.**
Limitations	19	Discuss limitations of the study, taking into account sources of potential bias or imprecision. Discuss both direction and magnitude of any potential bias	**9-10**	**See Discussion: Limitations.**
Interpretation	20	Give a cautious overall interpretation of results considering objectives, limitations, multiplicity of analyses, results from similar studies, and other relevant evidence	**7-10**	**See Discussion.**
Generalisability	21	Discuss the generalisability (external validity) of the study results	**9-10**	**See Discussion: Limitations.**
**Other information**				
Funding	22	Give the source of funding and the role of the funders for the present study and, if applicable, for the original study on which the present article is based	**Title page**	

Figure S1: Flow chart outlining inclusions, exclusions, and final sample size.

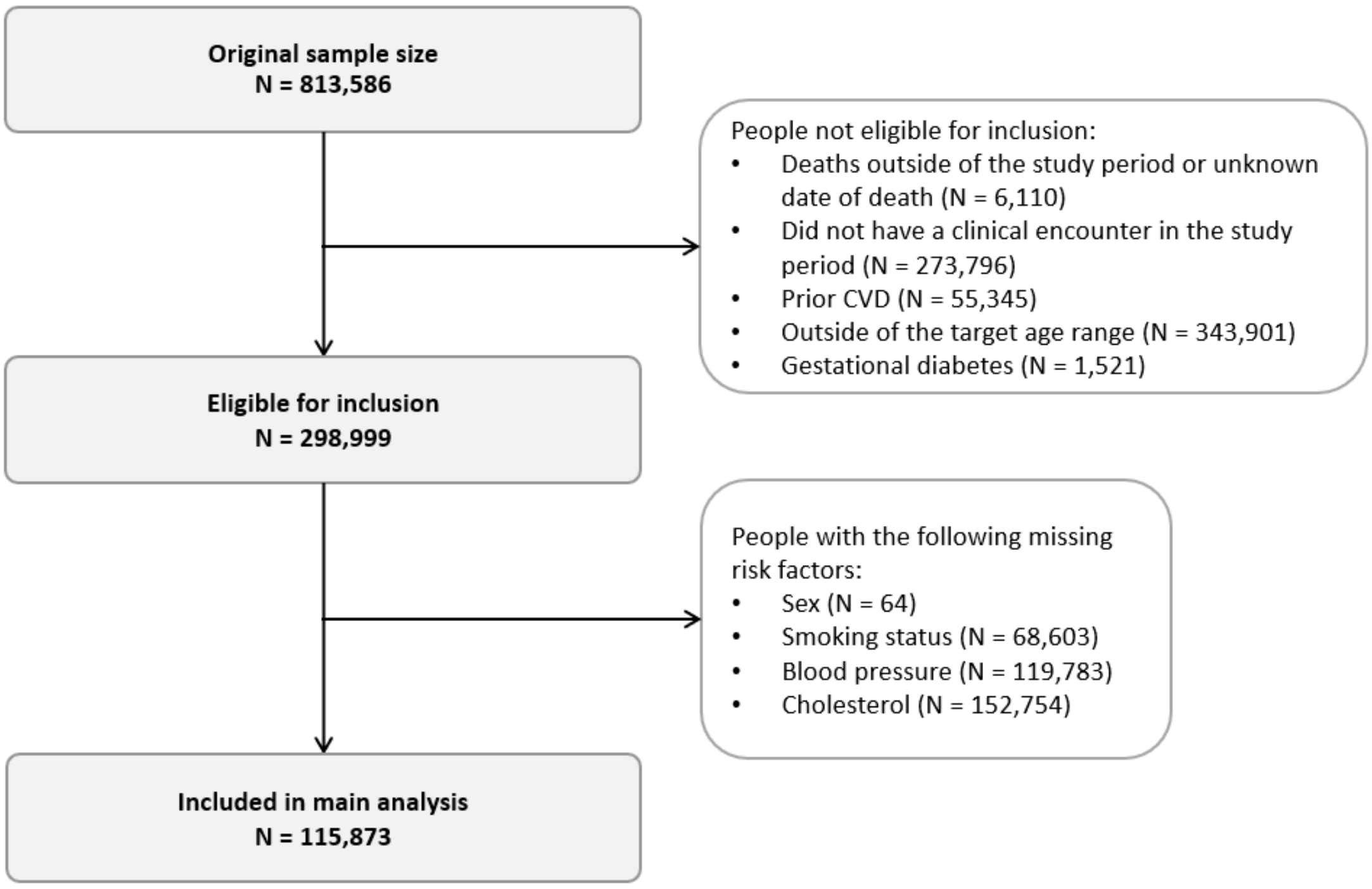

Table S3: Summary statistics for diabetes-specific risk factors among people with type-2 diabetes.

	Female	Male	Overall
**Number of people^**	5814	6700	12,514
**Years since diagnosed with diabetes mellitus**			
Median (IQR)	6.0 (2.0–11)	5.0 (2.0–10)	5.0 (2.0–11)
Missing N (%)	106 (1.8%)	111 (1.7%)	217 (1.7%)
**Haemoglobin A1c (mmol/mol)**			
Median (IQR)	51 (44–61)	52 (45–62)	52 (45–62)
Missing N (%)	180 (3.1%)	192 (2.9%)	372 (3.0%)
**Urinary albumin to creatinine ratio**			
Median (IQR)	1.2 (0.70–2.6)	1.2 (0.70–3.4)	1.2 (0.70–3.0)
Missing N (%)	1274 (21.9%)	1279 (19.1%)	2553 (20.4%)
**Estimated glomerular filtration rate (mL/min/1.73m^2)**			
Median (IQR)	88 (74–90)	89 (75–90)	88 (75–90)
Missing N (%)	216 (3.7%)	282 (4.2%)	498 (4.0%)
**Body mass index (kg/m^2)**			
Median (IQR)	33 (28–38)	31 (28–35)	32 (28–36)
Missing N (%)	1090 (18.7%)	1286 (19.2%)	2376 (19.0%)
**Use of insulin-related medication**			
No	5092 (87.6%)	5847 (87.3%)	10,939 (87.4%)
Yes	722 (12.4%)	853 (12.7%)	1575 (12.6%)

^Includes people who had clinically determined high risk.

Table S4: Distribution of selected risk factors among people who were not included in the analysis due to missing sex, smoking status, blood pressure, or cholesterol values.

	Overall
**Number of people**	183,126
**Sex**	
Female	100,551 (54.9%)
Male	82,511 (45.1%)
Missing	64 (0.0%)
**Age group (years)**	
45-54	75,756 (41.4%)
55-64	62,236 (34.0%)
65-74	45,134 (24.6%)
**Aboriginal and/or Torres Strait Islander person**	
No or not recorded	180,193 (98.4%)
Yes	2933 (1.6%)
**Socioeconomic quintile (SEIFA—IRSD, 2016)**	
1 (most disadvantaged)	29,963 (16.4%)
2	38,000 (20.8%)
3	36,641 (20.0%)
4	36,623 (20.0%)
5 (least disadvantaged)	40,730 (22.2%)
Missing	1169 (0.6%)
**Diabetes mellitus type 2**	
No	177,057 (96.7%)
Yes	6069 (3.3%)

Table S5: Distribution of selected risk factors among people who had missing and complete diabetes-specific risk factors among people with type-2 diabetes.

	Complete	Missing	Overall
**Number of people^**	7635	4437	12,072
**Aboriginal and/or Torres Strait Islander person**			
No	7338 (96.1%)	4287 (96.6%)	11,625 (96.3%)
Yes	297 (3.9%)	150 (3.4%)	447 (3.7%)
**Sex**			
Female	3502 (45.9%)	2100 (47.3%)	5602 (46.4%)
Male	4133 (54.1%)	2337 (52.7%)	6470 (53.6%)
**Age groups (years)**			
45-54	1575 (20.6%)	1022 (23.0%)	2597 (21.5%)
55-64	2800 (36.7%)	1645 (37.1%)	4445 (36.8%)
65-74	3260 (42.7%)	1770 (39.9%)	5030 (41.7%)
**Socioeconomic quintile (SEIFA—IRSD, 2016)**			
1 (most disadvantaged)	1677 (22.0%)	1033 (23.3%)	2710 (22.4%)
2	1890 (24.8%)	909 (20.5%)	2799 (23.2%)
3	1679 (22.0%)	910 (20.5%)	2589 (21.4%)
4	1286 (16.8%)	755 (17.0%)	2041 (16.9%)
5 (least disadvantaged)	1071 (14.0%)	799 (18.0%)	1870 (15.5%)
Missing	32 (0.4%)	31 (0.7%)	63 (0.5%)

^Excludes people who had clinically determined high risk.

Figure S2: Risk classification under the 2023 and 2012 algorithms, by sex and age category.

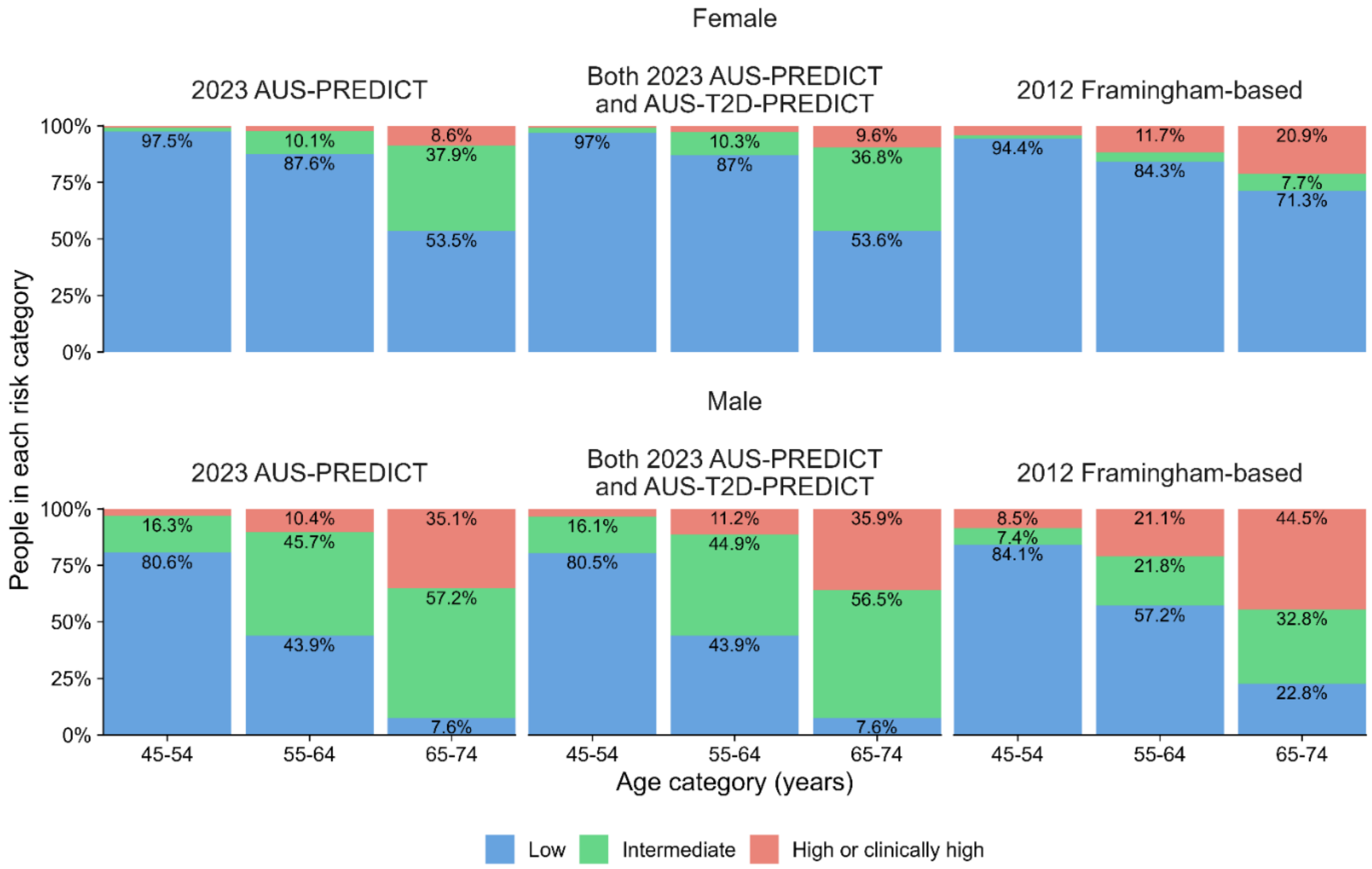

Note: AUS-PREDICT and AUS-T2D-PREDICT refer to the risk equations used in the 2023 Australian cardiovascular disease risk guidelines for the general population and people with type-2 diabetes, respectively.

Figure S3: Risk classification under the 2023 algorithms when including all people eligible (ages 45-79 years, 30-79 for Aboriginal and Torres Strait Islander people, and 35-79 years for people with diabetes).

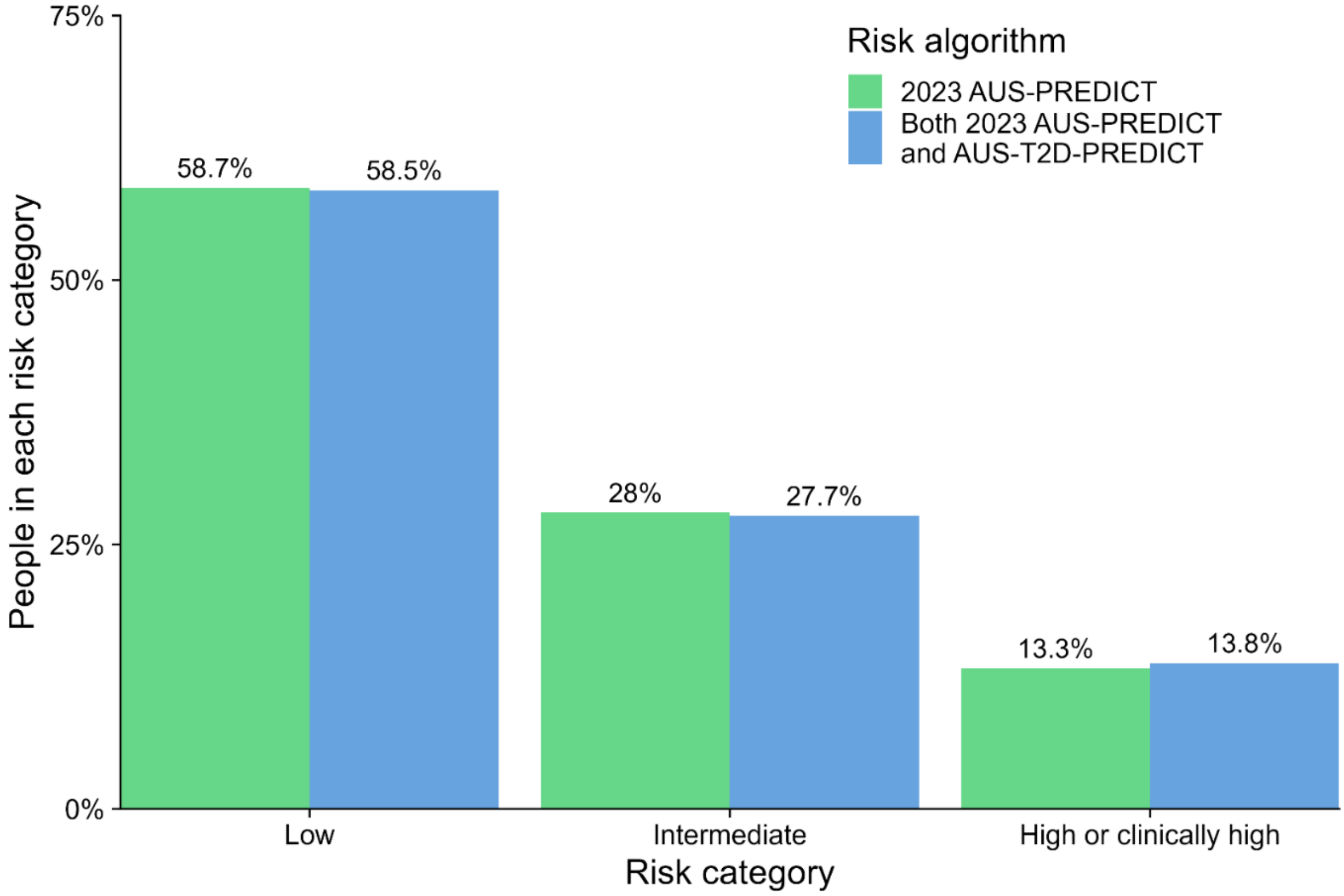

Note: AUS-PREDICT and AUS-T2D-PREDICT refer to the risk equations used in the 2023 Australian cardiovascular disease risk guidelines for the general population and people with type-2 diabetes, respectively.

Table S6: Risk classification under the 2023 and 2012 algorithms, overall and by sex, using the AUS-PREDICT general equation regardless of diabetes status. Row (R) and column (C) percentages are shown beneath cell frequencies.

Rows: **2023 AUS-PREDICT algorithm (general)**; Columns: **2012 Framingham-based algorithm**

OVERALL	Low	Intermediate	High	Clinically High	Total
**Low**	69,731	1,396	74	3,085	74,286
	(R:93.9%) (C:85%)	*(R:1.9%) (C:10.4%)*	*(R:0.1%) (C:1.2%)*	*(R:4.2%) (C:21.7%)*	*(R:100%) (C:64.1%)*
**Intermediate**	11,674	10,641	2,758	5,957	31,030
	(R:37.6%) (C:14.2%)	*(R:34.3%) (C:79.3%)*	*(R:8.9%) (C:44.5%)*	*(R:19.2%) (C:42%)*	*(R:100%) (C:26.8%)*
**High**	660	1,378	3,366	3,567	8,971
	(R:7.4%) (C:0.8%)	*(R:15.4%) (C:10.3%)*	*(R:37.5%) (C:54.3%)*	*(R:39.8%) (C:25.1%)*	*(R:100%) (C:7.7%)*
**Clinically High**	0	0	0	1,586	1,586
	(R:0%) (C:0%)	*(R:0%) (C:0%)*	*(R:0%) (C:0%)*	*(R:100%) (C:11.2%)*	*(R:100%) (C:1.4%)*
**Total**	82,065	13,415	6,198	14,195	115,873
	(R:70.8%) (C:100%)	*(R:11.6%) (C:100%)*	*(R:5.3%) (C:100%)*	*(R:12.3%) (C:100%)*	*(R:100%) (C:100%)*

FEMALE	Low	Intermediate	High	Clinically High	Total
**Low**	47,281	653	35	2,539	50,508
	(R:93.6%) (C:89.7%)	*(R:1.3%) (C:24%)*	*(R:0.1%) (C:5.7%)*	*(R:5%) (C:35.6%)*	*(R:100%) (C:79.9%)*
**Intermediate**	5,065	1,788	335	3,089	10,277
	(R:49.3%) (C:9.6%)	*(R:17.4%) (C:65.8%)*	*(R:3.3%) (C:54.8%)*	*(R:30.1%) (C:43.3%)*	*(R:100%) (C:16.3%)*
**High**	364	278	241	663	1,546
	(R:23.5%) (C:0.7%)	*(R:18%) (C:10.2%)*	*(R:15.6%) (C:39.4%)*	*(R:42.9%) (C:9.3%)*	*(R:100%) (C:2.4%)*
**Clinically High**	0	0	0	851	851
	(R:0%) (C:0%)	*(R:0%) (C:0%)*	*(R:0%) (C:0%)*	*(R:100%) (C:11.9%)*	*(R:100%) (C:1.3%)*
**Total**	52,710	2,719	611	7,142	63,182
	(R:83.4%) (C:100%)	*(R:4.3%) (C:100%)*	*(R:1%) (C:100%)*	*(R:11.3%) (C:100%)*	*(R:100%) (C:100%)*

MALE	Low	Intermediate	High	Clinically High	Total
**Low**	22,450	743	39	546	23,778
	(R:94.4%) (C:76.5%)	*(R:3.1%) (C:6.9%)*	*(R:0.2%) (C:0.7%)*	*(R:2.3%) (C:7.7%)*	*(R:100%) (C:45.1%)*
**Intermediate**	6,609	8,853	2,423	2,868	20,753
	(R:31.8%) (C:22.5%)	*(R:42.7%) (C:82.8%)*	*(R:11.7%) (C:43.4%)*	*(R:13.8%) (C:40.7%)*	*(R:100%) (C:39.4%)*
**High**	296	1,100	3,125	2,904	7,425
	(R:4%) (C:1%)	*(R:14.8%) (C:10.3%)*	*(R:42.1%) (C:55.9%)*	*(R:39.1%) (C:41.2%)*	*(R:100%) (C:14.1%)*
**Clinically High**	0	0	0	735	735
	(R:0%) (C:0%)	*(R:0%) (C:0%)*	*(R:0%) (C:0%)*	*(R:100%) (C:10.4%)*	*(R:100%) (C:1.4%)*
**Total**	29,355	10,696	5,587	7,053	52,691
	(R:55.7%) (C:100%)	*(R:20.3%) (C:100%)*	*(R:10.6%) (C:100%)*	*(R:13.4%) (C:100%)*	*(R:100%) (C:100%)*

Table S7: Risk classification under the 2023 algorithm for people with type-2 diabetes only, overall and by sex, comparing the AUS-PREDICT general equation and the AUS-T2D-PREDICT equation that includes additional (diabetes-specific) risk factors. Row (R) and column (C) percentages are shown beneath cell frequencies.

2023 AUS-T2D-PREDICT algorithm	2023 AUS-PREDICT algorithm				
OVERALL	**Low**	**Intermediate**	**High**	**Clinically High**	**Total**
**Low**	1,245 *(R:82.3%) (C:43.8%)*	268 *(R:17.7%) (C:4.5%)*	0 *(R:0%) (C:0%)*	0 *(R:0%) (C:0%)*	1,513 *(R:100%) (C:12.1%)*
**Intermediate**	482 *(R:14.3%) (C:17%)*	2,515 *(R:74.7%) (C:42.4%)*	370 *(R:11%) (C:11.2%)*	0 *(R:0%) (C:0%)*	3,367 *(R:100%) (C:26.9%)*
**High**	36 *(R:1.3%) (C:1.3%)*	977 *(R:35.5%) (C:16.5%)*	1,742 *(R:63.2%) (C:52.9%)*	0 *(R:0%) (C:0%)*	2,755 *(R:100%) (C:22%)*
**Clinically High**	0 *(R:0%) (C:0%)*	0 *(R:0%) (C:0%)*	0 *(R:0%) (C:0%)*	442 *(R:100%) (C:100%)*	442 *(R:100%) (C:3.5%)*
**Missing T2D risk factor/s**	1,078 *(R:24.3%) (C:37.9%)*	2,176 *(R:49%) (C:36.7%)*	1,183 *(R:26.7%) (C:35.9%)*	0 *(R:0%) (C:0%)*	4,437 *(R:100%) (C:35.5%)*
**Total**	2,841 *(R:22.7%) (C:100%)*	5,936 *(R:47.4%) (C:100%)*	3,295 *(R:26.3%) (C:100%)*	442 *(R:3.5%) (C:100%)*	12,514 *(R:100%) (C:100%)*
FEMALE	**Low**	**Intermediate**	**High**	**Clinically High**	**Total**
**Low**	1,059 *(R:85.7%) (C:45%)*	176 *(R:14.3%) (C:6.6%)*	0 *(R:0%) (C:0%)*	0 *(R:0%) (C:0%)*	1,235 *(R:100%) (C:21.2%)*
**Intermediate**	369 *(R:23.5%) (C:15.7%)*	1,128 *(R:72%) (C:42.4%)*	70 *(R:4.5%) (C:11.8%)*	0 *(R:0%) (C:0%)*	1,567 *(R:100%) (C:27%)*
**High**	29 *(R:4.1%) (C:1.2%)*	358 *(R:51.1%) (C:13.5%)*	313 *(R:44.7%) (C:52.8%)*	0 *(R:0%) (C:0%)*	700 *(R:100%) (C:12%)*
**Clinically High**	0 *(R:0%) (C:0%)*	0 *(R:0%) (C:0%)*	0 *(R:0%) (C:0%)*	212 *(R:100%) (C:100%)*	212 *(R:100%) (C:3.6%)*
**Missing T2D risk factor/s**	894 *(R:42.6%) (C:38%)*	996 *(R:47.4%) (C:37.5%)*	210 *(R:10%) (C:35.4%)*	0 *(R:0%) (C:0%)*	2,100 *(R:100%) (C:36.1%)*
**Total**	2,351 *(R:40.4%) (C:100%)*	2,658 *(R:45.7%) (C:100%)*	593 *(R:10.2%) (C:100%)*	212 *(R:3.6%) (C:100%)*	5,814 *(R:100%) (C:100%)*
MALE	**Low**	**Intermediate**	**High**	**Clinically High**	**Total**
**Low**	186 *(R:66.9%) (C:38%)*	92 *(R:33.1%) (C:2.8%)*	0 *(R:0%) (C:0%)*	0 *(R:0%) (C:0%)*	278 *(R:100%) (C:4.1%)*
**Intermediate**	113 *(R:6.3%) (C:23.1%)*	1,387 *(R:77.1%) (C:42.3%)*	300 *(R:16.7%) (C:11.1%)*	0 *(R:0%) (C:0%)*	1,800 *(R:100%) (C:26.9%)*
**High**	7 *(R:0.3%) (C:1.4%)*	619 *(R:30.1%) (C:18.9%)*	1,429 *(R:69.5%) (C:52.9%)*	0 *(R:0%) (C:0%)*	2,055 *(R:100%) (C:30.7%)*
**Clinically High**	0 *(R:0%) (C:0%)*	0 *(R:0%) (C:0%)*	0 *(R:0%) (C:0%)*	230 *(R:100%) (C:100%)*	230 *(R:100%) (C:3.4%)*
**Missing T2D risk factor/s**	184 *(R:7.9%) (C:37.6%)*	1,180 *(R:50.5%) (C:36%)*	973 *(R:41.6%) (C:36%)*	0 *(R:0%) (C:0%)*	2,337 *(R:100%) (C:34.9%)*
**Total**	490 *(R:7.3%) (C:100%)*	3,278 *(R:48.9%) (C:100%)*	2,702 *(R:40.3%) (C:100%)*	230 *(R:3.4%) (C:100%)*	6,700 *(R:100%) (C:100%)*

Figure S4: Distributions of cardiovascular disease risk estimates from the 2023 and 2012 risk equations. Noting that the two 2023 risk estimate distributions (in blue and green) lie on top of each other.

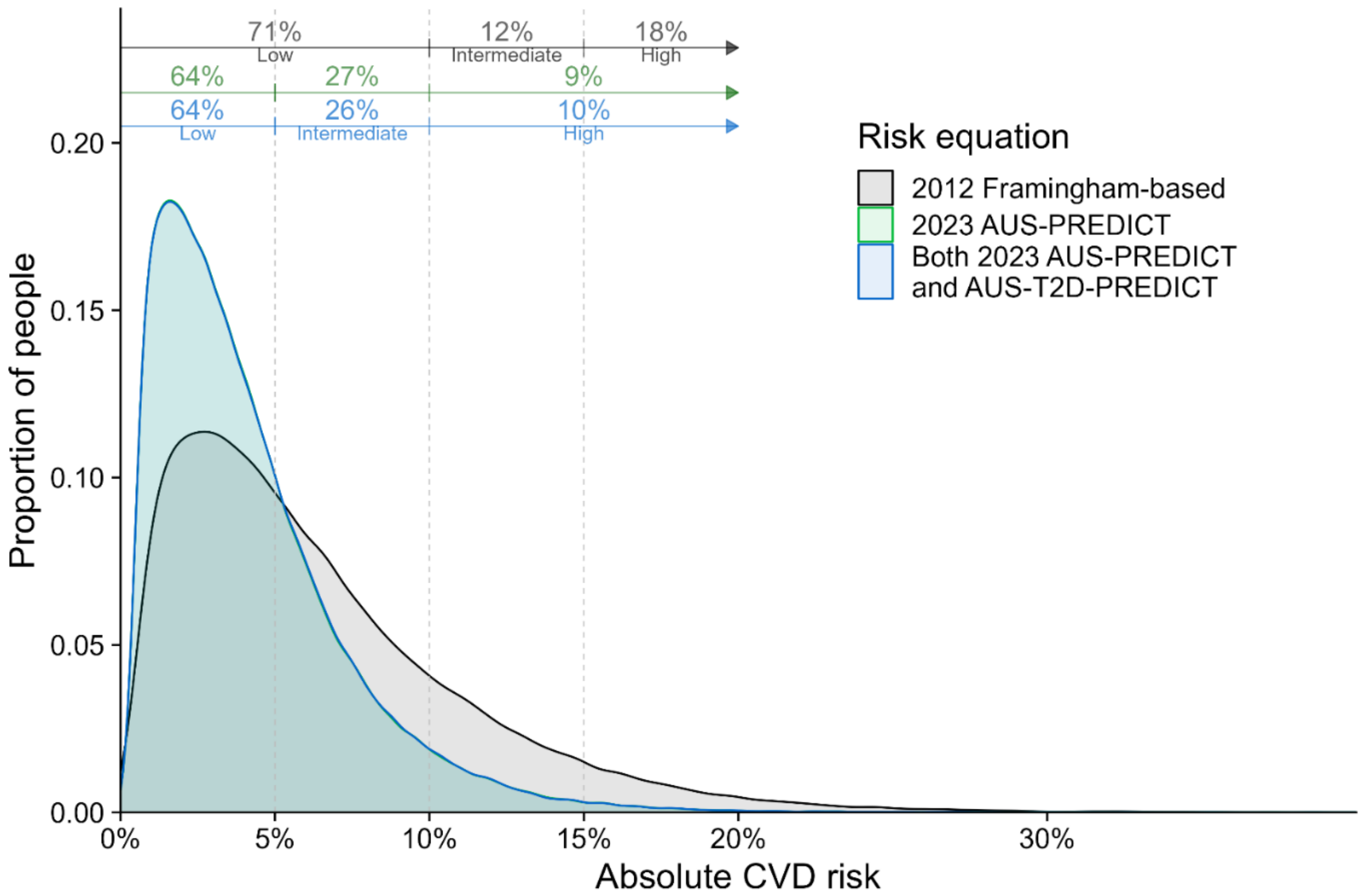

Note: The proportions in each risk category include people who were at clinically determined high risk. AUS-PREDICT and AUS-T2D-PREDICT refer to the risk equations used in the 2023 Australian cardiovascular disease risk guidelines for the general population and people with type-2 diabetes, respectively.

Figure S5A and S5B: Distributions of cardiovascular disease risk estimates from the 2023 and 2012 risk equations, for females (A) and males (B). Noting that the two 2023 risk estimates distributions (in blue and green) lie on top of each other.

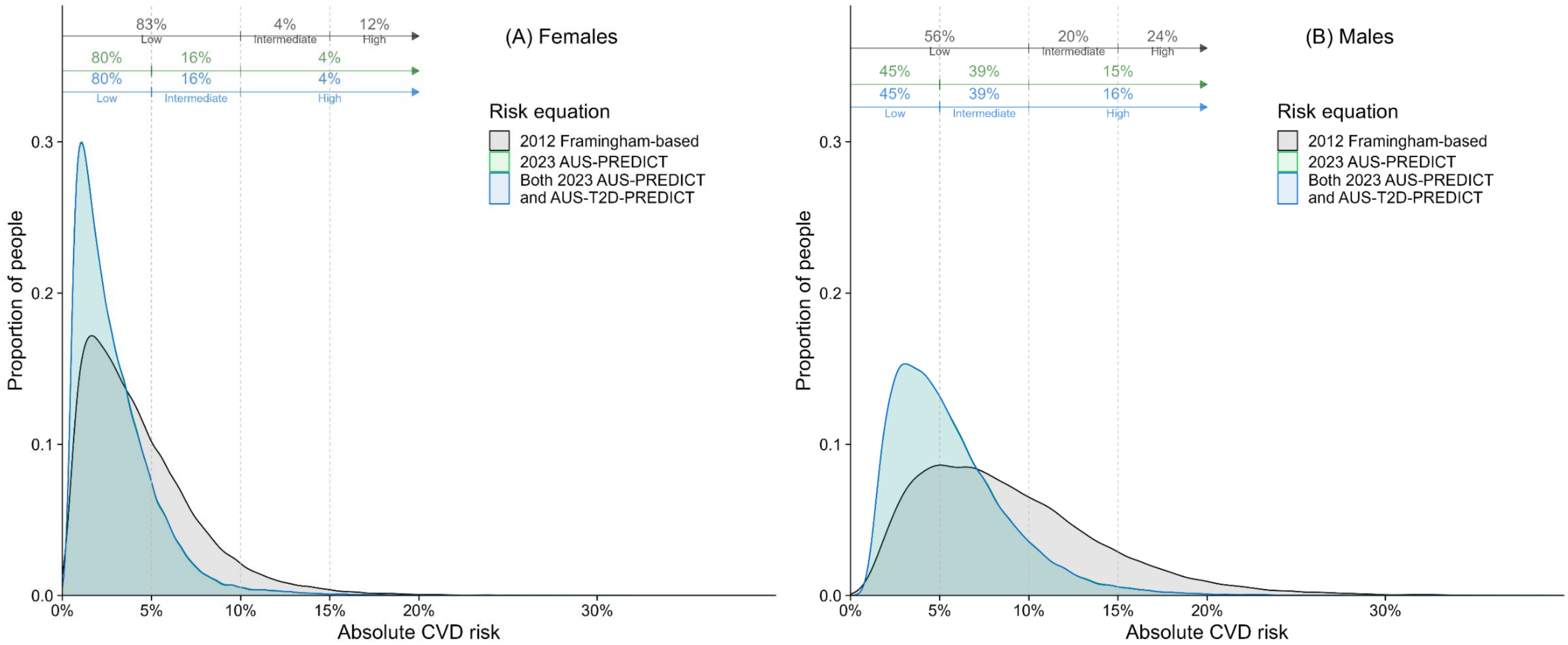

Note: The proportions in each risk category include people who were at clinically determined high risk. AUS-PREDICT and AUS-T2D-PREDICT refer to the risk equations used in the 2023 Australian cardiovascular disease risk guidelines for the general population and people with type-2 diabetes, respectively.

Figure S6A and S6B: Bland-Altman plots of 2023 AUS-PREDICT (including AUS-T2D-PREDICT for patients with type-2 diabetes) versus 2012 Framingham-based cardiovascular disease risk estimates. Panel (A) shows the differences in risks and Panel (B) shows the difference in log risks (i.e., ratio of risks), versus the mean cardiovascular disease risk across equations from the two guidelines. Both plots show the mean difference and the lower and upper 95% limits of agreement (LOA).

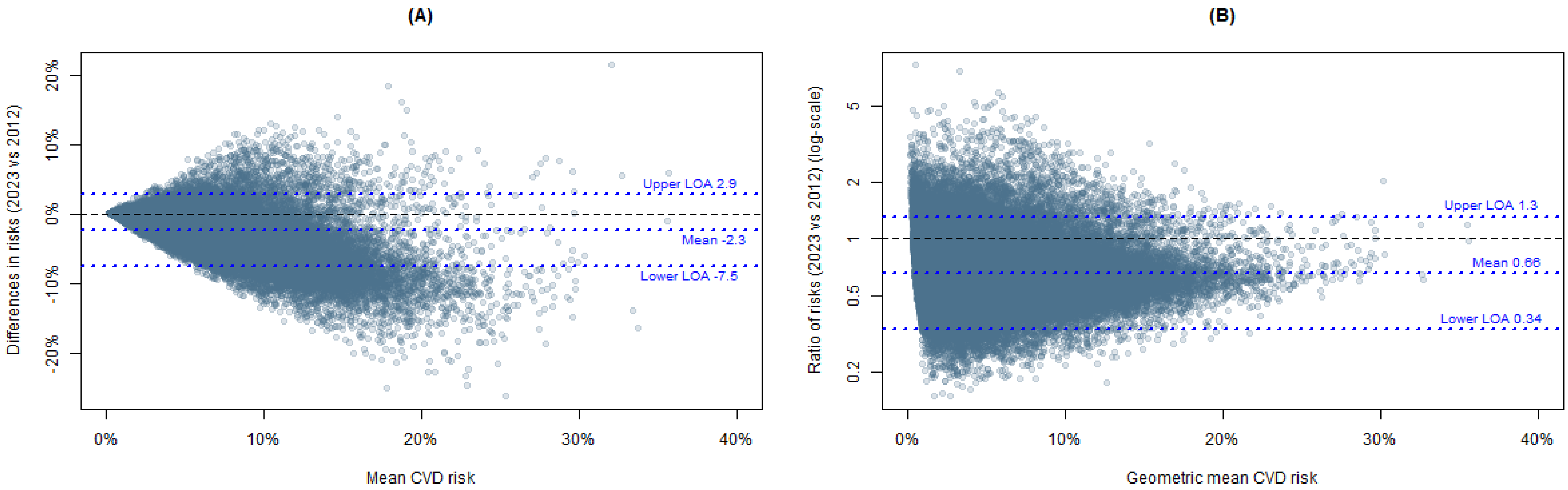

Figure S7A and S7B: Bland-Altman plots of 2023 AUS-PREDICT (including AUS-T2D-PREDICT for patients with type-2 diabetes) versus 2012 Framingham-based cardiovascular disease risk estimates for females. Panel (A) shows the differences in risks and Panel (B) shows the difference in log risks (i.e., ratio of risks), versus the mean cardiovascular disease risk across equations from the two guidelines. Both plots show the mean difference and the lower and upper 95% limits of agreement (LOA).

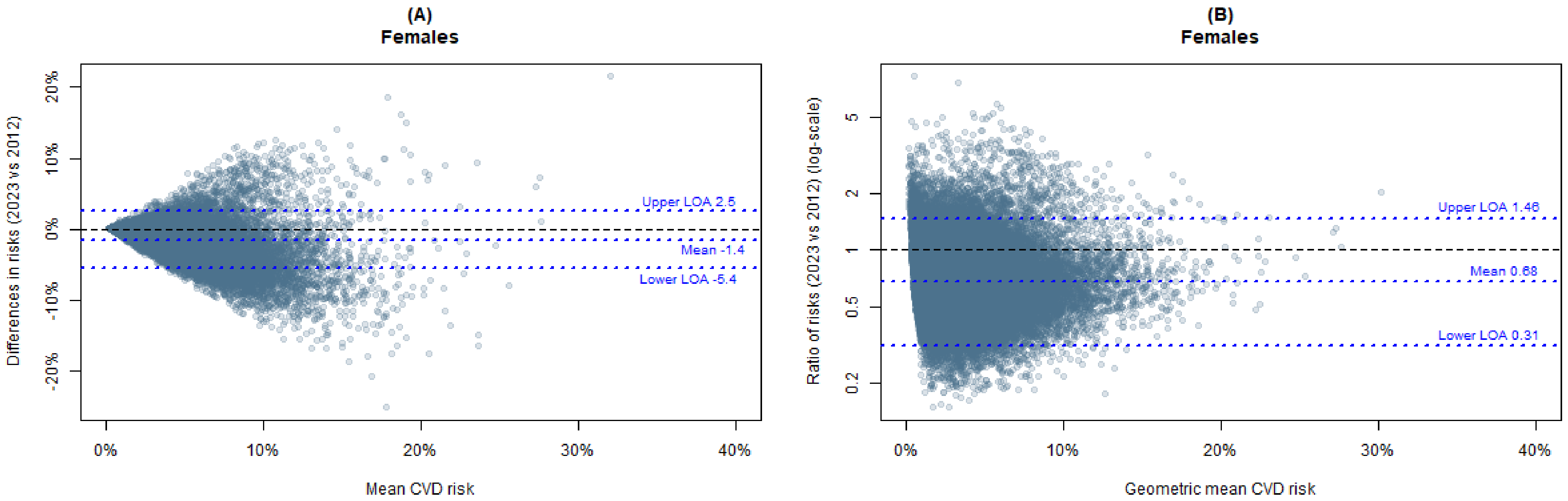

Figure S8A and S8B: Bland-Altman plots of 2023 AUS-PREDICT (including AUS-T2D-PREDICT for patients with type-2 diabetes) versus 2012 Framingham-based cardiovascular disease risk estimates for males. Panel (A) shows the differences in risks and Panel (B) shows the difference in log risks (i.e., ratio of risks), versus the mean cardiovascular disease risk across equations from the two guidelines. Both plots show the mean difference and the lower and upper 95% limits of agreement (LOA).

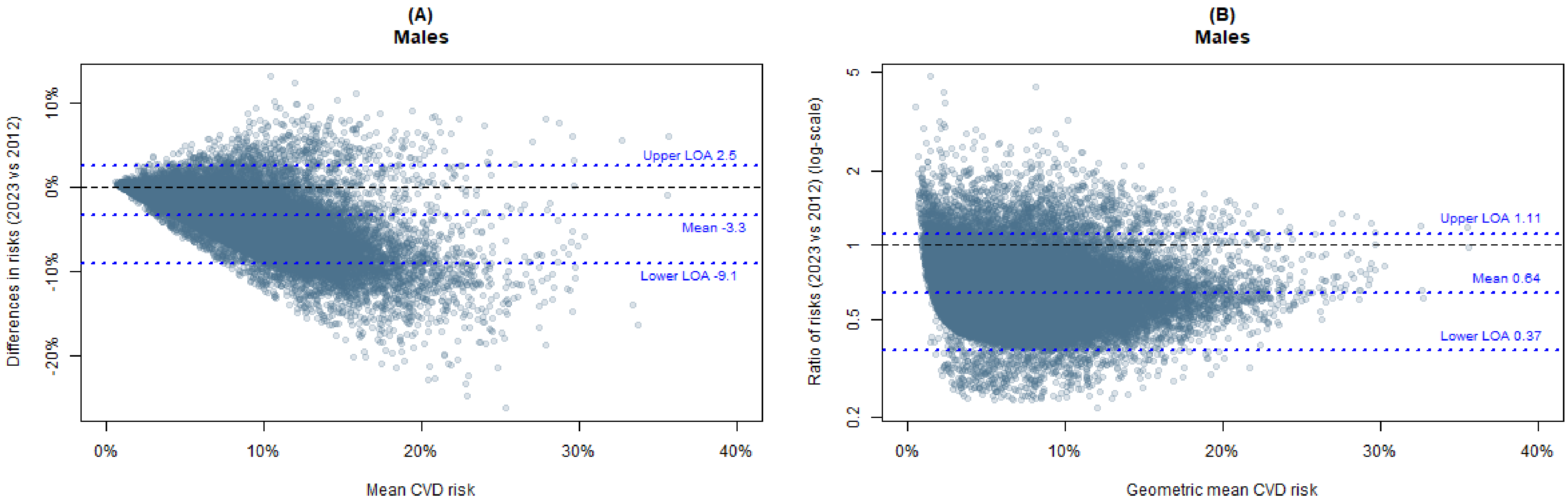

Figure S9A and S9B: Bland-Altman plots of cardiovascular disease risk estimates from AUS-T2D-PREDICT versus AUS-PREDICT for people with type-2 diabetes. Panel (A) shows the differences in risks and Panel (B) shows the difference in log risks (i.e., ratio of risks), versus the mean cardiovascular disease risk across the two equations. Both plots show the mean difference and the lower and upper 95% limits of agreement (LOA).

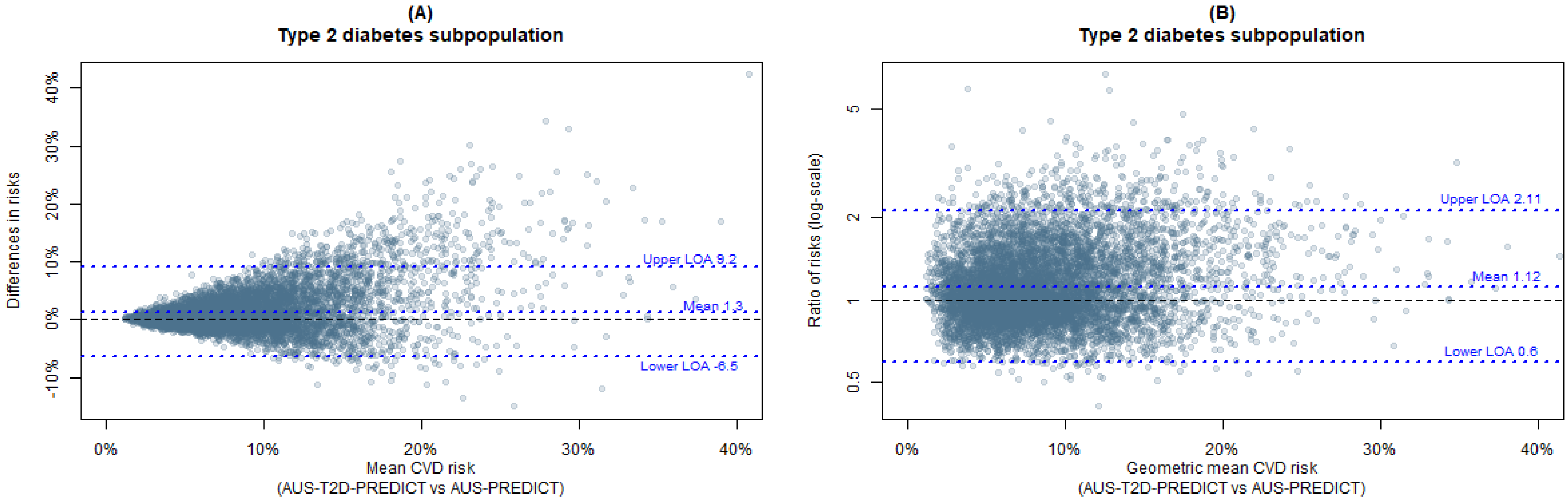

References

1. Australian Bureau of Statistics. 2033.0.55.001 - Census of Population and Housing: Socio-Economic Indexes for Areas (SEIFA): Index of Relative Socio-economic Disadvantage (IRSD) (ABS, 2016). Available from: https://www.abs.gov.au/ausstats/abs@.nsf/Lookup/by%20Subject/2033.0.55.001~2016~Main%20Features~IRSD~19 (viewed May 2026)

2. Havard A, Manski-Nankervis JA, Thistlethwaite J, et al. Validity of algorithms for identifying five chronic conditions in MedicineInsight, an Australian national general practice database. *BMC Health Services Research* 2021; 21: 551.

3. Bandyopadhyay D, Qureshi A, Ghosh S, et al. Safety and efficacy of extremely low LDL-cholesterol levels and its prospects in hyperlipidemia management. *Journal of Lipids* 2018; 8598054.

4. Rader DJ and deGoma EM. Approach to the patient with extremely low HDL-cholesterol. *Journal of Clinical Endocrinology and Metabolism*, 2012; 97(10): 3399-407.
